# Air pollution exposure in relation to guard duty at Tidworth Camp: A cross-sectional study

**DOI:** 10.1371/journal.pone.0258070

**Published:** 2021-09-30

**Authors:** Ngwa Niba Rawlings, Akwah Emmanuela Ambe, Lem Ngongalah

**Affiliations:** 1 Defence Medical Academy, Defence Medical Services (DMS), Whittington, United Kingdom; 2 Leeds Beckett University, Leeds, United Kingdom; 3 The Collaboration for Research Excellence in Africa (CORE Africa), Douala, Cameroon; Universidade Federal do Rio Grande - FURG, BRAZIL

## Abstract

**Background:**

Air pollution is the largest environmental health risk in the United Kingdom, and an issue of concern amongst outdoor workers. Road transport is a major source producing the largest amount of nitrogen dioxide (NO_2_) and ozone (O_3_) (as a secondary pollutant). Hundreds of vehicles enter and exit the Tidworth Camp’s main gate daily, potentially producing these pollutants. However, the air pollution exposure experienced by personnel on guard duty is unknown. This study aimed to determine and compare background NO_2_ and O_3_ levels experienced by personnel on guard duty.

**Methods:**

Cross-sectional data was collected using a static sampling technic on randomly selected days of the week. Data analysis was done using IBM-SPSS-26 and a *p*-value of <0.05 was considered statistically significant.

**Results:**

The background concentration of NO_2_ and O_3_ pollutants were within recommended limits. There was no significant difference between mean morning and afternoon exposure levels for both pollutants. However, NO_2_ and O_3_ levels were significantly higher during weekdays compared to weekends (M = -0.022, SD = 0.007, t(6) = -8.672, *p* <0.0001 and M = -0.016, SD = 0.008, t(6) = -5.040, *p* = 0.002 respectively). Both pollutants showed no significant differences in exposure levels when only weekdays were compared. NO_2_ levels showed a weak positive correlation during weekdays (*r* = 0.04) and a strong positive correlation during weekends (*r* = 0.96). O_3_ levels had a positive correlation on both weekdays and weekends; however, levels on Monday showed a negative correlation (*r* = -0.55). Linear regression analysis showed that outside temperature was a significant predictor of O_3_ levels (*p* = 0.026).

**Conclusion:**

Personnel on guard duty experienced higher pollution levels during weekdays compared to weekends; however, air pollution levels for both pollutants were within recommended limits. Further studies are recommended over hotter months using a personal sampling technic to measure personal air pollution exposure levels in order to minimise any health and safety risks.

## Introduction

Air pollution is a common issue worldwide and is the largest environmental health risk in the United Kingdom (UK) [1]. In 2016, 91% of the global population lived in polluted areas, and out door air pollution caused 4.2 million premature deaths [2]. According to the European Environment Agency (EEA), Nitrogen dioxide (NO_2_) and Ozone (O_3_) were amongst the top three air pollutants producing the most serious health effects to humans and deaths in Europe [3]. People from lower socio-economic groups are the most exposed; while the elderly, children and those with underlying medical conditions are more susceptible [4]. NO_2_ and O_3_ are two main air pollutants of concern in the UK, with road transport being the main source, causing both environmental and health effects [4–7]. Health effects include diseases such as asthma, lung cancer, heart disease and stroke [2]. A study by the British Safety Council showed that 36,000 early deaths occur every year from outdoor air pollution in the UK, with 9,400 premature deaths in London alone [8].

The European Union (EU) Ambient Air Quality Directive (2008/50/EC) established legal limits for concentrations of major air pollutants (including NO_2_, and O_3_), and the UK adopted these limits into the Air Quality Standards Regulations 2010, which is monitored regularly. The Health and Safety Executive (HSE) published a document (EH40/2005 Workplace exposure limits—WELs) which contains British occupational exposure limits, with the aim of protecting health by ensuring that people are not exposed to harmful quantities of hazardous substances in the workplace [9]. The HSE’s EH40 workplace exposure limit for NO_2_ (8-hr TWA) is 0.96 mg/m^3^ and for O_3_ (15-min TWA) is 0.4mg/m^3^ [9]. However, there is a growing concern about health effects from outdoor air pollution in the UK, with recommended limits being exceeded. For example, in 2019, 76.7% (33 out of 43) monitoring zones exceeded the limit for annual mean NO_2_ air pollution in the UK [7]. In a report published by The Royal College of Physicians, people who work near busy roads (such as traffic police, street cleaners, road maintenance workers, and security guards) were identified as one of the most vulnerable groups [10]; and are at highest risk of exposure to unhealthy levels of air pollution. Several studies have shown an increase in different health effects as a result of NO_2_ and O_3_ pollution [11–16]. Occupational exposure to outdoor air pollution have been reported amongst commercial drivers of buses, cars, and motorcycles [17].

Tidworth camp is part of the Tidworth, Netheravon and Bulford garrison (TidNBul) and one of the largest military garrison in the UK, located in the Southwest Region of England. It is home to more than 15000 military and civilian personnel. Hundreds of vehicles (including small cars, buses, and armoured trucks) enter and exit the Tidworth Camp’s main gate daily. Personnel on guard duty carry out security checks on all these vehicles as they drive into the Camp. These vehicles could be seen queuing-up at the main gate during busier hours (mornings– 7:00 to 9:00 am, launch time– 11:00 am to 13:00 pm and afternoons– 15:00 to 17:00 pm); thereby increasing the risk of air pollution exposure to personnel on guard duty. No studies have been conducted on military bases in the UK to determine air pollution exposure experienced by personnel on guard duty despite the variety of military vehicles entering and exiting military Camps. This study aimed to determine background workplace contaminant concentrations of NO_2_ and O_3_ exposure, and to compare the daily exposure levels experienced by personnel on guard duty at the Tidworth Camp’s main gate.

## Methods

Tidworth Camp is part of the Tidworth, Netheravon and Bulford garrison located in the Southwest Region of England and hosts thousands of military and civilian personnel. The number of personnel is expected to increase due to an influx of troops withdrawn from Germany [18]. The study was conducted in February 2021 using a cross-sectional design. NO_2_ and O_3_ levels were collected using the AQY1-Micro Air Quality Monitor [19]. Data collection was done on randomly selected days–three days were selected using a computer-generated simple random selection tool (Monday and Tuesday to represent weekdays, and Sunday to represent weekends).

Data validity was achieved through equipment calibration and setup following the manufacturer’s instructions [20]. Two-hourly NO_2_ and O_3_ data (from 7:00am to 19:00pm) were extracted from the equipment and double checked for any errors before analysis. The two-hourly data was selected to mimic the guard duty shift pattern. The extracted data was then entered into IBM SPSS version 26 for further analysis. Data analysis involved calculating means and standard deviation to provide a summary of the data set; t-test to compare exposure levels; correlation analyses to check for any existing relationships and a regression analyses to identify which variables had an impact on the air pollution level.

### Ethics

This study was guided by the Helsinki Declaration as revised in 2013. The study did not involve any human participants; however, ethical approval was provided by Leeds Beckett University ethics committee. A letter of permission (Gate keeper letter) was also provided by the Tidworth Camp for the study. As the study was conducted during the COVID-19 period, all UK government guidance on COVID-19 social distancing were observed.

## Results

Table 1 shows the descriptive statistics for the study. The daily maximum and minimum temperatures were 10.5°C and -1.41°C and an average maximum and minimum wind speed of 3.1m/s and 0.9m/s, respectively. NO_2_ and O_3_ exposure levels varied from day to day partly due to variability in weather conditions (mostly windspeed) and traffic flow. Standard deviations (SD) showed that the two-hourly exposure levels for each day did not deviate much from the daily mean. The mean exposure level for NO_2_ and O_3_ was highest on Monday and Tuesday respectively; while Sunday had the lowest mean exposure level for both pollutants.

**Table 1:** Daily NO_2_ and O_3_ exposure levels.

Time (2 hrly)	Sunday			Monday			Tuesday		
	NO_2_ (mg/m^3^)	O_3_ (mg/m^3^)	Temp (°C)	NO_2_ (mg/m^3^)	O_3_ (mg/m^3^)	Temp (°C)	NO_2_ (mg/m^3^)	O_3_ (mg/m^3^)	Temp (°C)
07:00	0.0400	0.0306	0.87	0.0599	0.0565	-1.41	0.0601	0.0526	-1.15
09:00	0.0408	0.0341	2.59	0.0700	0.0602	-0.46	0.0683	0.0501	-1
11:00	0.0412	0.0456	10.5	0.0649	0.0609	0.01	0.0665	0.0559	-0.54
13:00	0.0441	0.0466	7.8	0.0693	0.0558	-0.08	0.0718	0.0554	0.2
15:00	0.0442	0.0528	7.18	0.0675	0.0555	-0.81	0.0645	0.0692	0.89
17:00	0.0478	0.0408	4.45	0.0710	0.0564	-0.85	0.0700	0.0628	-0.8
19:00	0.051	0.0378	2.99	0.0591	0.0554	-1.1	0.06	0.0562	-0.95
**Mean**	0.0442	0.0412	5.20	0.0660	0.0572	-0.67	0.0659	0.0575	-0.48
**SD**	0.004	0.0077	3.42	0.0048	0.0023	0.52	0.0046	0.0065	0.75
**WS**	0.9m/s			2.8m/s			3.1m/s		

**Note:** SD = standard deviation; Temp = temperature; °C = degree Celsius; WS = average daily wind speed; mg/m^3^ = milligrams per metres cube; hrly = hourly.

**Fig 1:**
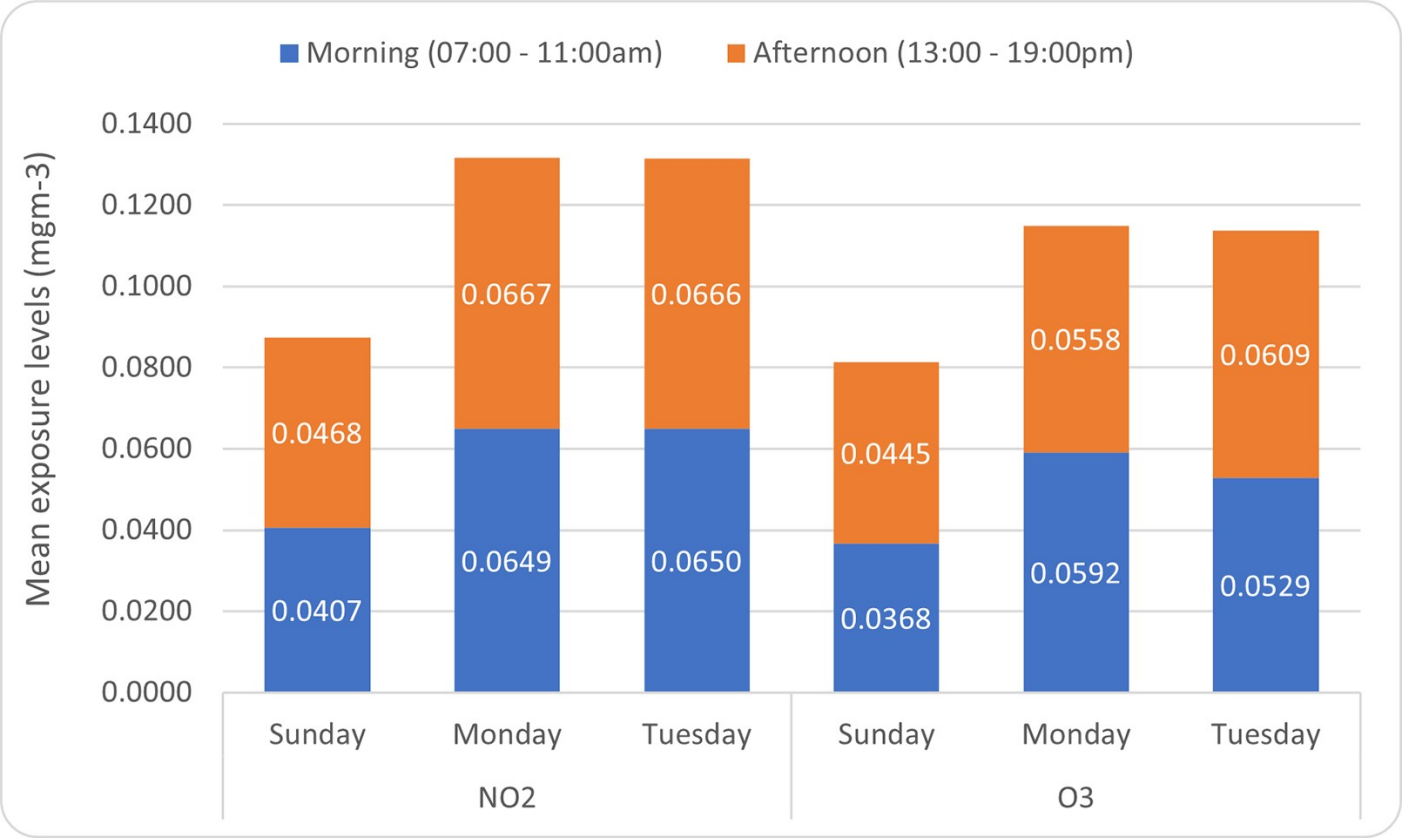
Average NO_2_ and O_3_ exposure levels.

Paired t-test analysis showed that there were no significant differences between the average morning (am) and afternoon (pm) exposure levels for both pollutants. However, exposure levels were significantly higher during weekdays (Monday and Tuesday) compared to weekends (Sunday) for both pollutants. No significant difference was found in exposure levels for both pollutants when only weekdays (Monday and Tuesday) were compared. See Table 2 and Fig 1 below.

**Table 2:** Paired sample t-test analysis.

Paired Samples	Mean	Std. Deviation	Std. Error Mean	95% Confidence Interval		t	df	*p* value
				Lower	Upper			
NO_2_ Sun (am)—NO_2_ Sun (pm)	-.0047000	.0016823	.0009713	-.0088790	.0000210	-4.839	2	.060
NO_2_ Mon (am)—NO_2_ Mon (pm)	-.0043333	.0061436	.0035470	-.0195948	.0109281	-1.222	2	.346
NO_2_ Tue (am)—NO_2_ Tue (pm)	-.0038000	.0077544	.0044770	-.0230629	.0154629	-.849	2	.485
O_3_ Sun (am)—O_3_ Sun (pm)	-.0099667	.0128594	.0074244	-.0419111	.0219778	-1.342	2	.312
O_3_ Mon (am)—O_3_ Mon (pm)	.0033000	.0022539	.0013013	-.0022990	.0088990	2.536	2	.127
O_3_ Tue (am)—O_3_ Tue (pm)	-.0096000	.0084788	.0048952	-.0306625	.0114625	-1.961	2	.189
NO_2_ Sun—NO_2_ Mon	-.0218000	.0066513	.0025140	-.0279514	-.0156486	-8.672	6	.00013*
NO_2_ Sun—NO_2_ Tue	-.0217286	.0064376	.0024332	-.0276823	-.0157748	-8.930	6	.00011*
NO_2_ Mon—NO_2_ Tue	.0000714	.0019371	.0007322	-.0017201	.0018630	.098	6	.925
O_3_ Sun—O_3_ Mon	-.0160571	.0084295	.0031860	-.0238531	-.0082612	-5.040	6	.002*
O_3_ Sun—O_3_ Tue	-.0162714	.0051919	.0019623	-.0210731	-.0114697	-8.292	6	.00017*
O_3_ Mon—O_3_ Tue	-.0002143	.0078671	.0029735	-.0074901	.0070616	-.072	6	.945
NO_2_ Sun—O_3_ Sun	.0029714	.0081061	.0030638	-.0045255	.0104683	.970	6	.370
NO_2_ Mon—O_3_ Mon	.0087143	.0049181	.0018589	.0041658	.0132628	4.688	6	.003*
NO_2_ Tue—O_3_ Tue	.0084286	.0077448	.0029273	.0012658	.0155913	2.879	6	.028*

**Note:** am–morning; pm–afternoon; Sun–Sunday; Mon–Monday; Tue–Tuesday; t–t-test; df–degree of freedom

*significant *p*-value of <0.05.

**Fig 2:**
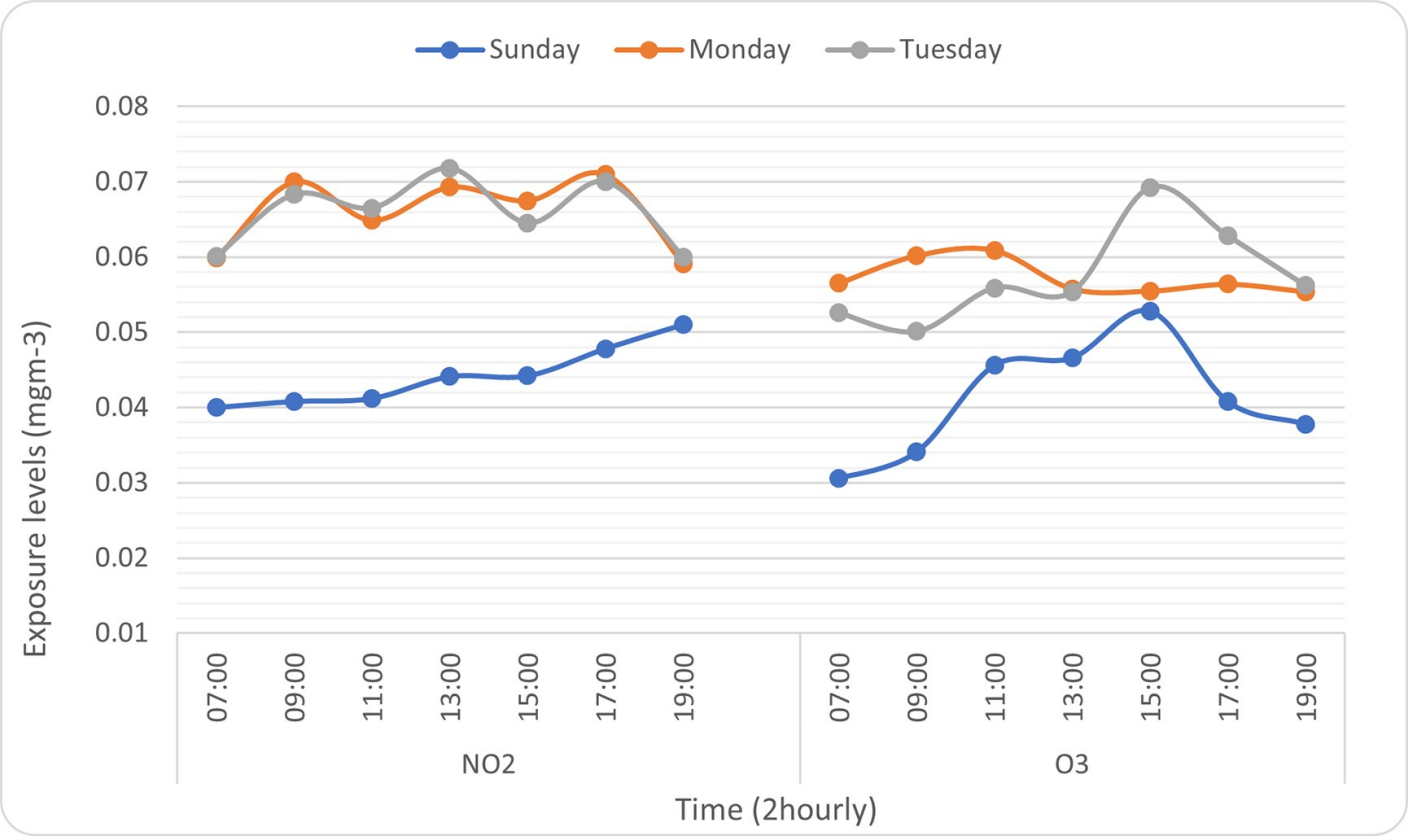
NO_2_ and O_3_ levels showing weekly correlation.

NO_2_ exposure levels had a strong positive correlation during weekends (Sunday–*r* = 0.96), and a weak positive correlation during weekdays (Monday–*r* = 0.04 and Tuesday–*r* = 0.02). O_3_ exposure levels had a strong positive correlation during weekends (Sunday–r = 0.42) and weekdays (Tuesday–r = 0.59); however, O_3_ levels had a strong negative correlation on Monday (r = -0.55). See Fig 2.

A paired sample correlation analysis was done to determine the relation between NO_2_ level and outside temperature (T^o^) (independent variables–IV) and O_3_ level (dependant variable–DV). The results showed that both NO_2_ and O_3_ levels had weak positive correlations (*r* = 0.16, *r* = 0.20, and *r* = 0.06), while T^o^ and O_3_ level had a stronger positive correlation (*r* = 0.84, *r* = 0.55, and *r* = 0.71) on Sunday, Monday, and Tuesday, respectively. A linear regression analysis was performed to predict O_3_ level (DV) from NO_2_ level (IV_1_) and T^o^ (IV_2_). A significant regression equation was observed {F(2, 4) = 6.22, *p* = 0.026), with an *R*^*2*^ of 0.757. The predicted O_3_ level was 0.011 + 0.002 (T^o^) (T^o^ measured in °C). O_3_ level increased 0.002mg/m^-3^ for each °C of T^o^. Outside temperature was a significant predictor of O_3_ level (*p* = 0.026), while NO_2_ level was not (*p* = 0.397) (see Table 3).

**Table 3:** Effect of NO_2_ level and outside temperature on O_3_ levels.

Independent variable	Coefficient
Outside temperature	.002 (.001)*
NO_2_ level	.450 (.474)
Constant	.011
R	.870
R Square	.757
Adjusted R Square	.635
F-ratio	6.22*
n	**7**

Note

* = p < 0.05; coefficients are unstandardised with standard errors in brackets.

## Discussion

The aim of this study was to determine background workplace contaminant concentrations of NO_2_ and O_3_, and to compare the daily exposure levels experienced by personnel on guard duty at the Tidworth camp’s main gate. Exposure levels were first compared to check for differences between morning (07:00–11:00 am) and afternoon exposure (13:00–19:00 pm). The mean afternoon exposure for both pollutants was marginally higher than the mean morning exposure levels; however, the difference was not significant. Similar results have been reported in other studies where afternoon exposure levels were higher than morning exposure levels, but with no statistically significant differences [7,21]. However, some studies have also found contradictory results, with significantly higher pollution exposure levels during morning periods [22]. Higher exposures during working hours increases the risk to health of personnel.

When the mean daily NO_2_ and O_3_ exposure levels for weekdays and weekends were compared, the results showed significant higher exposure levels for both pollutants during weekdays. The differences in exposure levels during weekdays and weekends were consistent with findings from an air quality report by Defra, showing a peak in NO_2_ pollution levels during weekdays, with concentrations being 20% greater compared to weekends [23]. As was the case in this study, Defra attributed this difference to high traffic seen during weekdays compared to weekends. This implies that personnel on guard duty may be exposed to higher pollution levels and are at higher risk of developing health effect during weekdays compared to weekends. No significant difference in mean exposure levels were observed for both pollutants when only weekdays were compared.

Correlation analyses showed a direct proportional relationship between NO_2_ exposure levels and daily hours on weekends; that is NO_2_ levels had a strong positive correlation during weekends. However, during weekdays, NO_2_ exposure levels showed a weak positive correlation. The weak positive correlation could be seen to exhibit spikes in NO_2_ levels at specific times of the day (usually busier hours). These spikes were observed at 7:00–9:00, 11:00–13:00 and 15:00–17:00; and these hours represented periods of high traffic at the Camp’s main gate as personnel went to work (7:00–9:00); went for lunch breaks (11:00–13:00); and went home after work (15:00–17:00). These findings are consistent with those of a report by Defra showing high air pollution levels during morning and evening rush hours, as a result of traffic congestion [24].

O_3_ showed a positive correlation on both weekdays and weekends, but exposure levels on one of the weekdays (Monday) showed a negative correlation which could be attributed to the very low temperatures observed on that day (see Table 1). As seen from this findings and in line with a report by Defra [23], the amount of O_3_ produced is dependent on the amount of NO_2_ and temperature available. Our findings were consistent with those of a similar study that showed a positive correlation of daily averaged O_3_ with air temperature [25].

Linear regression analysis showed a strong relationship between the T^o^ and O_3_ level. From the adjusted R square value obtained, 63.5% of the variance in O_3_ level could be attributed to T^o^. The linear regression analysis model showed that T^o^ was a significant predictor of O_3_ level while NO_2_ level was not a significant predictor of O_3_ level. This implies that personnel on guard duty during hotter days are potentially exposed to high amounts of O^3^ and are at high risk of its health effects. Other studies have shown similar findings [25]; however our findings contradicted those of a study showing NO_2_ as a predictor of O_3_ level [22].

Overall, the exposure levels of both pollutants were low and within recommended levels [9]. However, because this study used a static sampling technic and not a personal sampling technic to collect data; therefore, results could not be directly compared to the workplace exposure limits provided by the Health and Safety Executive (HSE). Nonetheless, the exposure levels obtained from this study could be used as a baseline background workplace exposure level because the HSE has recommended static sampling as a suitable technics for determining background workplace contaminant concentrations [26].

This study had the following limitations: personal sampling technic could not be used due to COVID-19 social distancing measures; the study was conducted over COVID-19 period when a small amount of traffic entered and exited the Camp (as many personnel were working from home) and did not reflect the actual traffic situation on a normal day; the study was conducted over the winter period (February) when weather conditions such as temperature, rainfall and wind speed is known to affect pollution levels [21].

## Conclusion

This study analysed background workplace contaminant concentrations of NO_2_ and O_3_ exposure levels and compared the daily exposure levels experienced by personnel on guard duty at the Tidworth Camps’ main gate. The results showed that the mean NO_2_ and O_3_ exposure levels for all days measured were within recommended levels. When the NO_2_ and O_3_ exposure levels were compared, the results showed no significant difference between mean morning and mean afternoon exposure levels for both pollutants. However, the mean exposure levels for both pollutants were significantly higher during weekdays compared to weekends; implying that personnel on guard duty were exposed to higher levels of air pollution during weekdays. During weekdays, NO_2_ exposure levels increased with high traffic at busier hours. Outside temperature was the only significant predictor of O_3_ levels. While this study provided background air pollution levels for personnel on guard duty at the Tidworth Camp’s main gate and daily trends in exposure levels which were unknown, it is recommended that further studies be conducted using personal sampling technic, over hotter months and on a larger scale and the results compared with those of this study.
